# Recognition of stroke-related knowledge among community residents and the improvement after intensive health education: a cross-sectional study

**DOI:** 10.1186/s12883-020-01951-6

**Published:** 2020-10-12

**Authors:** Xuemin Zhong, Jian Wang, Lanying He, Ronghua Xu

**Affiliations:** Department of Neurology, The Second People’s Hospital of Chengdu, Chengdu, Sichuan China

**Keywords:** Stroke, Community residents, Warning symptoms, Intensive education

## Abstract

**Background:**

The high morbidity, mortality, and disability rates of stroke constitute a heavy burden to the society. The level of understanding about stroke warning symptoms and first aid systems among community residents was generally low. The aim of our work is to investigate stroke-related knowledge in community residents of Jinjiang district of Chengdu and to raise public awareness about stroke through an intensive educational program.

**Methods:**

Nine communities in Jinjiang district were sampled and a questionnaire about stroke-related knowledge was applied before and after stroke educational activities. We analyzed the impact of such activities in stroke recognition and management.

**Results:**

We collected 1685 valid questionnaires. The awareness about stroke risk before educational activities was 11.4%. The recognition of stroke warning signs among community residents was 29.8–59.5%. Among them, the recognition of major signs, such as limb weakness, language disorder, and imbalance was more than 50%. When faced with five stroke warning signs, the proportion of participants who chose to make an emergency call was 41.5%. Less than 10% of the participants chose to consult a doctor, take medicine, or wait. After strengthening publicity and educational activities regarding stroke, there was a significant improvement in the identification of stroke risk factors, warning signs, and stroke management. The percentage of participants who chose to make an emergency call increased from 53.2 to 82.7%.

**Conclusions:**

The knowledge about stroke among community residents in Jinjiang District of Chengdu was low. Strengthening publicity and educational activities may raise awareness about stroke prioritizing call emergency after the onset of stroke signs.

## Background

Stroke is a serious disease with the highest disability rate. Its high morbidity, mortality, and disability rates constitute a heavy burden and a great source of pain to the society, families, and patients [1]. The number of new stroke patients in China is about 2 million every year, of which 70–80% is unable to live independently due to disability [2]. At present, the incidence of stroke in China is increasing at an annual rate of 8.7% [3]. The nationwide annual cost for the treatment of cerebrovascular disease is more than 10 billion yuan, coupled with indirect economic losses of nearly 20 billion yuan [4]. Various health education measures have been undertaken to raise public awareness of stroke-related knowledge to reduce the incidence, mortality, and disability rates of stroke [5]. However, the level of understanding about stroke warning symptoms and first aid systems among community residents was generally low [6–8]. This study investigated the ability residents to identify the warning symptoms of stroke and the awareness of first aid and the effect of strengthening health education on stroke recognition among community residents. Herein, we aimed to investigate the current status and problems of community stroke prevention and to strengthen the publicity of the impact of stroke-related knowledge on community residents’ stroke recognition, to propose and implement a new community stroke prevention service model to effectively reduce community stroke incidence.

## Methods

### Sampling

We conducted a survey of residents of nine communities of Jinjiang District, Chengdu, from February 2017 to February 2019 (including Chunxi, Yanshikou, Niushikou, Hongsha, Jinjiang, Lianxin, Wanke, Quan subtree, Daci temple). According to the method of estimating the minimum sample size of qualitative data recommended by Chinese Residents of Nutrition and Health Survey in 2002, 1800 households in Jinjiang District, the center of Chengdu with a permanent population of 690,400, were randomly selected. A cluster sampling method was adopted, with sampling of 200 households in each community according to the residence number. The participants were permanent residents of the community (The inclusion criteria were aged ≥18; household registration was required to be considered a local, and the residents should have lived in the locality for more than 2 years).

### Survey contents

Using an epidemiological investigation, combined with the literature reports from China and abroad [1–3, 5–9], a questionnaire on stroke-related knowledge was designed to conduct a cluster sampling survey among nine community residents in Jinjiang District, Chengdu. The questionnaire had 21 questions, which contained 4 main sections:

1. Respondents’ demographic details such as sex, age, ethnicity, educational level, monthly household income, and health insurance.
2. Respondents’ understanding of stroke risk factors (including high blood pressure, hyperlipidemia, diabetes, heart disease, drinking, smoking, stroke, obesity, age, genetic factors, atherosclerosis, vascular stenosis, lack of exercise, blood viscosity and etc).
3. Respondents’ recognition of stroke warning signs [5 “Suddens,” including (i) sudden difficulty in speaking, understanding, or slurred speech, (ii) sudden blurred vision in one or both eyes, (iii) sudden severe headache with unknown cause, (iv) sudden dizziness, difficulty in walking, loss of balance or co-ordination, and (v) sudden numbness or weakness of the face and/or limb(s) on one side of the body]. The method of rapid identification of stroke involves applying the acronym “FAST” which means numbness or weakness on one side of the Face, numbness or weakness in the Arm, Speech or understanding difficulties and Time to call).
4. Respondents’ awareness of the first-aid system to sudden symptoms of stroke. Respondents were asked the questions: If you are sure that someone is having a stroke, what will your first reaction be? (i) drive to hospital, (ii) call for doctor, (iii) call for 120; (iv) call for family. We aimed to carry out stroke health education activities with the theme of “understanding stroke” and evaluate the effect. Stroke knowledge was publicized in a variety of ways, including: (1) designing and making short videos which neurologists explained the risk factors of stroke, its prevention and treatment, and the description of the main symptoms and signs of stroke. The session on the first aid after the onset of stroke emphasized the importance of making an emergency call (120, in China), and the harm of delayed treatment. Stroke survivors were also invited to talk about their personal experience. We used television, the Internet, WeChat, magazines, and other media channels for publicity. (2) We produced a pamphlet on stroke health education to be distributed to all families in the community, and produced posters of stroke-related knowledge to display in public places in the community. The duration of intensive stroke education activity was 1 year. All the participants received health information from all or some of the media mentioned. The media platforms contained the same health information. We provided information pamphlets to the participants and encouraged them to take the pamphlet home. The level of community stroke awareness was investigated before and after the activity, and the effect of intensive publicity was evaluated. The main evaluation indicators were the proportion of residents that accepted stroke-related knowledge due to the publicity, and the change of residents’ recognition of stroke-related knowledge (stroke risk factors, stroke symptoms and signs, stroke and the treatment of specific symptoms). Face-to-face interviews were conducted by uniformly trained investigators.

### Data collection

Data were collected via a questionnaire on community residents’ stroke-related knowledge. The contents mainly included: (1) the general data of the participants; (2) stroke-related knowledge before and after the intensive publicity: stroke risk factors, warning symptoms, treatment measures of sudden symptoms, determination of post-stroke treatment.

### Statistical analysis

After sorting out the data processing and survey data, the data were entered into the EPIDATA database and then imported into SPSS version 20 (IBM Corp., Armonk, NY, USA) for statistical analysis. Descriptive statistical analysis was used to assess the general characteristics of respondents, identify stroke-related risk factors, identify warning symptoms of stroke, and cope with stroke warning symptoms. Chi-square test was used to analyze the general characteristics of residents before and after intensive education and the residents’ knowledge of stroke-related knowledge before and after intensive education. *P* value less than 0.05 was regarded as statistically significant.

## Results

### Demographic characteristics of the respondents

A total of 1685 respondents completed the questionnaires, with the response rate of 93.6%. Of the 1685 respondents, 768 were males (45.6%) and 917 females (54.4%), with an average age of 50.5 ± 16.2 (range, 18–92) years. Demographic details are as shown in Table 1.

**Table 1:** Demographic data of the respondents (*N* = 1685)

	*Number of Respondents*	*%*
Gender		
Male	768	45.6
Female	917	54.4
Age (y)		
18–36	399	23.7
37–55	591	35.1
56–74	575	34.1
≥ 75	120	7.1
Ethnicity		
Han	1668	99.0
Other	17	1.0
Marital status		
Unmarried	196	11.6
Married or cohabiting	1347	79.9
Divorce or separation	54	3.2
Bereavement	88	5.2
Educational level		
Primary school or less	182	10.8
Middle school	416	24.7
High school/technical school	594	35.3
College or more	493	29.3
Health insurance		
Yes	1567	93.0
No	118	7.0
Smoke		
Yes	476	28.2
No	1209	71.8

### Relationship between the number of stroke risk factors and stroke risk recognition

The participants were assessed for the presence of risk factors (Table 2). The awareness level of stroke risk among community residents was 11.4%. Only 40.3% of participants with three or more risk factors were aware of the risk of stroke. One hundred and fifty-four participants (9.1%) could correctly identify 1 stroke risk factor, and 132 (7.8%) could correctly identify 2 stroke risk factors. One hundred and thirty-three participants (7.9%) were able to identify at least 3 stroke risk factors. Also, 1164 participants (69.1%) could not correctly identify any risk factors for stroke, of which 1061 cases (91.2, 63.0% of the total) could not provide any answer. Of the 1685 subjects, 922 (54.7%) that did not know which part of the body was affected by stroke, only 699 (41.5%) knew that stroke affected the brain, and 17 (1.0%) believed that stroke affected the heart. Forty-seven participants (2.8%) believed that stroke affected other parts (such as the hands, feet, cervical vertebrae, etc.).

**Table 2:** Cognition of stroke risk factors among respondents

Risk factor	N	(%)
High blood pressure	385	22.8
Hyperlipidemia	188	11.2
Diabetes	170	10.1
Heart disease	125	7.4
Unhealthy lifestyle	95	5.6
Drinking	90	5.3
Smoking	88	5.2
Stroke	72	4.3
Obesity	47	2.8
Age	31	1.8
Genetic factors	26	1.5
Atherosclerosis	19	1.1
Vascular stenosis	11	0.7
Lack of exercise	11	0.7
Blood viscosity	9	0.5
Gender	4	0.2
False		
Emotional	33	2.0
Overwork	20	1.2
Mental stimulation	10	0.6
Other	6	0.4

### Recognition of stroke warning symptoms among participants

The recognition rate of stroke warning symptoms among community residents was 29.8–59.5% (Table 3). Among them, the recognition rate of common stroke symptoms such as limb weakness, language disorder, and balance disorder, was more than 50%. For the other two relatively uncommon symptoms, the rate of recognition of severe headaches, including blurred monocular or binocular vision and no known cause, was only about 29.8%. In addition, more than 25% considered shortness of breath, chest pain, and panic as symptoms of stroke.

**Table 3:** Cognition of stroke warning symptoms

Warning symptoms	Yes	No	No idea or uncertain
Difficulty speaking, articulating, or understanding	953 (56.6)	149 (8.8)	583 (34.6)
Shortness of breath	470 (27.9)	380 (22.6)	835 (49.6)
Blurred vision in one eye or both eyes	502 (29.8)	336 (19.9)	847 (50.3)
Severe headache of unknown cause	612 (36.3)	208 (12.3)	865 (51.3)
Chest pain, panic	533 (31.6)	335 (19.9)	817 (48.5)
Dizziness, difficulty walking, imbalance, or uncoordinated movements	956 (56.7)	138 (8.2)	591 (35.1)
Numbness or weakness on one side of the face or limb	1002 (59.5)	119 (7.1)	564 (33.5)

### Recognition of stroke warning signs

When faced with five sudden stroke warning signs, the proportion of participants who chose to dial 120 was lower, and the proportion that chose to go to the hospital was similar to that of those who chose to dial 120. Nearly 10% of respondents chose others such as consulting a doctor, taking medicine, waiting, or observing (Table 4).

**Table 4:** Cognition of stroke warning signs management in subjects [n (%)]

Warning signs	Drive to hospital	Consulting a doctor	Call for 120	Call family members	Other
Difficulty speaking, articulating, or understanding	719 (42.7)	221 (13.1)	599 (35.5)	48 (2.8)	98 (5.8)
Shortness of breath	604 (35.8)	180 (10.7)	769 (45.6)	37 (2.2)	95 (5.6)
*Blurred vision in one eye or both eyes	577 (34.2)	270 (16.0)	680 (40.4)	66 (3.9)	92 (5.5)
*Severe headache of unknown cause	723 (42.9)	136 (8.1)	709 (42.1)	40 (2.4)	77 (4.6)
Chest pain, panic	652 (38.7)	167 (9.9)	702 (41.7)	45 (2.7)	119 (7.1)
*Dizziness, difficulty walking, imbalance, or uncoordinated movements	708 (42.0)	128 (7.6)	736 (43.7)	47 (2.8)	66 (3.9)
*Numbness or weakness on one side of the face or limb	745 (44.2)	117 (6.9)	697 (41.4)	47 (2.8)	79 (4.7)

*five sudden stroke warning signs

### Assessment after strengthening health education

Before intensive publicity, there were 1685 respondents that completed the questionnaires. After intensive publicity, there were 1617 respondents that completed the questionnaires. There was no significant difference in the general situation of residents’ cognition of stroke before and after intensive publicity and education (Table 5). After the onset of a stroke, the only appropriate response is to immediately activate emergency medical services (call 120, in China). After strengthening publicity and education, there was a significant improvement in the identification of stroke risk factors, stroke warning symptoms, and stroke management. The number of respondents who would dial 120 increased from 53.2 to 82.7% (Table 6).

**Table 5:** General situation of residents before and after intensive publicity

	Before (*n* = 1685)	After (*n* = 1617)	*P*
Gender			0.059
Male	594	688	
Female	920	929	
Age (mean ± SD)	51.2 ± 15.6	53.4 ± 16.6	0.289
Nationality			0.493
Ethnic Han	1500	1598	
Other	14	19	
Marital status			0.418
Unmarried	190	199	
Married or cohabiting	1147	1258	
Divorce or separation	67	62	
Bereavement	110	98	
Degree			0.274
Primary schools and below	178	201	
Junior middle school	397	407	
High school	605	689	
College or above	334	320	
Medical insurance			0.803
Yes	1407	1499	
No	107	118	
Smoke			0.306
Yes	487	548	
No	1027	1069

**Table 6:** Cognition of Stroke-related knowledge before and after intensive educational activities

	Before (*n* = 1685)	After (*n* = 1617)	*P*
List the number of risk factors for stroke	2.62 ± 1.46	4.18 ± 2.07	< 0.001
Answer risk factors ≥3 (%)	628 (41.5)	1009 (62.4)	< 0.001
List the number of stroke warning signs	1.28 ± 1.25	1.67 ± 1.37	< 0.001
Know stroke warning symptoms ≥2	542 (37.3)	779 (48.2)	< 0.001
Answer 5 Suddens correctly	138 (8.2)	269 (16.6)	< 0.001
Answer “FAST” correctly	119 (7.1)	306 (18.9)	< 0.001
Call for 120	896 (53.2)	1338 (82.7)	< 0.001

## Discussion

The results of this study showed that the awareness level of stroke risk among community residents was 11.4%. Only 40.3% of participants with three or more risk factors were aware of the risk of stroke. The results suggested that community residents had a low level of awareness of stroke risk. Among all the subjects, 1164 participants (69.1%) could not correctly identify any risk factors for stroke, of which 1061 cases (91.2, 63.0% of the total) did not provide any answer. However, a foreign demographic survey conducted in 2015 showed that 59.2% of the respondents were able to identify at least one stroke risk factor [10]. This study concluded that the community residents in Jinjiang District had a low awareness of stroke risk factors. Similar to some foreign survey, it is necessary to strengthen the popularity of the knowledge regarding stroke [11].

Of the 1685 participants, 922 (54.7%) did not know which part of the body was affected by stroke, only 699 (41.5%) knew that stroke affected the brain. Similar to the report of a foreign study, 35% of respondents in this study knew that the organ in which stroke occurs was the brain [12]. These results suggest that community residents have poor knowledge of stroke-related symptoms.

The recognition rate of stroke warning symptoms among community residents was 29.8–59.5%. Among them, the recognition rate of common stroke symptoms such as limb weakness, language disorder, and balance disorder, was more than 50%. For the other two relatively uncommon symptoms, the recognition rate of severe headaches, including blurred monocular or binocular vision and no known cause, was only about 29.8%. In addition, more than 25% considered shortness of breath, chest pain, and panic as stroke manifestations, suggesting that community residents still lack knowledge of stroke warning symptoms. A domestic survey on the recognition of stroke among community residents in Chongqing found that the recognition rate of stroke warning symptoms was 30.7–75.6% [9]. A study of 1472 respondents found that sudden headaches (54.1%), vertigo (51.0%), and dyslexia (44.3%) were the most recognizable stroke warning symptoms [13].

When faced with five sudden stroke warning symptoms, most of the respondents chose to send the patients to hospital emergency department (by dialing 120 or going to the hospital themselves), but the proportion who preferred dialing 120 was lower. Similar to foreign reports, a survey conducted in New York found that 33.3–72.4% [14] of emergency calls were first made in the face of stroke warning symptoms, compared with 14–17.6% in Michigan [15]. The results of the latest questionnaire survey on a small sample size abroad showed that 73.0% of the respondents chose to go to the hospital themselves and emergency calls comprised 43.8 and 62.6% [16], respectively. Calling the emergency system in time allows patients to arrive at the hospital more quickly and receive diagnosis and treatment (such as stroke green channels such as recombinant tissue plasminogen activator) than if they were sent to the hospital or via other transport routes.

In this study, we designed and produced television short films, animated short films, and distributed health pamphlets to explain the risk factors of stroke and their prevention and treatment measures. Through 1 year of intensive publicity and education, the residents’ recognition of stroke-related knowledge (stroke risk factors, stroke symptoms and signs, stroke and the treatment of specific symptoms) was significantly improved. The number of respondents who dialed the 120-emergency system increased from 53.2% before publicity to 82.7%. This study believes that strengthening publicity and education of stroke-related knowledge, and post-stroke treatment can significantly improve the cognition of stroke-related knowledge and enhance the community residents’ awareness of the priority of dialing 120 after the onset of stroke. It can effectively reduce the pre-hospital delay in patients with stroke.

The shortcomings of this study are as follows: (1) the scope of the survey is limited to Jinjiang District of Chengdu, the sample size was small, and the results do not reflect the overall situation of Chengdu; (2) compared with an open-ended questionnaire, the closed questions used in this survey may result in a higher response rate in the evaluation of cognitive ability of stroke-related knowledge; and (3) the failure to ensure consistency of residents before and after participation in the intensive education.

## Conclusions

In summary, the results of this community-based survey showed that the recognition of stroke warning symptoms of community residents in our city is low. In the face of sudden symptoms of stroke, even among those who have cognitive ability to all common stroke warning symptoms, the proportion that choose to dial 120 was also lower. Taking measures to further improve the recognition of stroke-related knowledge among community residents can enhance the awareness of community residents to call 120 after the onset of stroke and effectively reduce the pre-hospital delay among stroke patients.

## Data Availability

The datasets used and/or analyzed during the current study are available from the corresponding author on reasonable request.
